# Responses of Neurons in Lateral Intraparietal Area Depend on Stimulus-Associated Reward During Binocular Flash Suppression

**DOI:** 10.3389/fnsys.2019.00009

**Published:** 2019-03-12

**Authors:** Hamed Bahmani, Qinglin Li, Nikos K. Logothetis, Georgios A. Keliris

**Affiliations:** ^1^Department of Physiology of Cognitive Processes, Max Planck Institute for Biological Cybernetics, Tuebingen, Germany; ^2^Bernstein Center for Computational Neuroscience, Tuebingen, Germany; ^3^Division of Imaging Science and Biomedical Engineering, University of Manchester, Manchester, United Kingdom; ^4^Bio-Imaging Lab, Department of Biomedical Sciences, University of Antwerp, Wilrijk, Belgium

**Keywords:** LIP, BFS, reward, electrophysiology, visual perception, parietal cortex

## Abstract

Discovering neural correlates of subjective perception and dissociating them from sensory input has fascinated neuroscientists for a long time. Bistable and multistable perception phenomena have exhibited great experimental potential to address this question. Here, we performed electrophysiological recordings from single neurons in lateral intraparietal area (LIP) of rhesus macaques during stimulus and perceptual transitions induced by binocular flash suppression (BFS). LIP neurons demonstrated transient bursts of activity after stimulus presentation and stimulus or perceptual switches but only a minority of cells demonstrated stimulus and perceptual selectivity. To enhance LIP neural selectivity, we performed a second experiment in which the competing stimuli were associated with asymmetric rewards. We found that transient and sustained activities substantially increased while the proportion of stimulus selective neurons remained approximately the same, albeit with increased selectivity magnitude. In addition, we observed mild increases in the proportion of perceptually selective neurons which also showed increase magnitude of selectivity. Importantly, the increased selectivity of cells after the reward manipulation was not directly reflecting the reward size *per se* but an enhancement in stimulus differentiation. Based on our results, we conjecture that LIP contributes to perceptual transitions and serves a modulatory role in perceptual selection taking into account the stimulus behavioral value.

## Introduction

Neural correlates of visual awareness have been attracting scientists’ interest for several decades. Bistable and multistable perception phenomena demonstrated great experimental potential to address this question (Attneave, 1971; Rock et al., 1994; Rock, 1995; Leopold and Logothetis, 1999; Logothetis, 1999). A unique paradigm proven to be invaluable for the study of perceptual organization is binocular rivalry (BR; Logothetis, 1998). During BR, sufficiently dissimilar images are presented to the two eyes in homologous retinal positions and lead to spontaneous and stochastic perceptual alternations between each monocular view (Leopold and Logothetis, 1999). Since these perceptual transitions occur in the absence of changes in the external stimuli, neural responses associated to these transitions and the perception of the alternating stimuli can be dissociated from pure sensory processing of external stimulus characteristics and changes in the input. Several single-cell recording and imaging studies have shown an increasing correlation between the neural activity and subjective perception during BR while moving up in visual hierarchy. However, this has mainly been done in the ventral visual stream (Logothetis, 1998; Leopold and Logothetis, 1999; Keliris et al., 2010). In stark contrast to the ventral stream, evidence for the neural correlates of perceptual transitions in the dorsal stream has only been studied so far in humans that highlighted a central role of the fronto-parietal network in perceptual changes during BR and bi-stable views (Lumer et al., 1998; Knapen et al., 2011). Thus, single cell studies are necessary in order to understand the relationships between both the previous single cell studies in the ventral stream as well as the human findings that were based on indirect functional imaging measurements.

A limitation present in the study of perceptual organization in the dorsal stream is the weak or no selectivity of neurons to basic visual stimulus features such as contours, color, spatial frequency *et cetera* defining object identity. Moreover, responses in these areas and in particular the lateral intraparietal area (LIP) that has been postulated as a homolog of human parietal areas, claimed to be involved in perceptual alternations (Orban, 2016), are modulated by attention, reward, perceptual decisions and other cognitive variables. To this end, saliency has been suggested as a unifying explanation for LIP activity (Bisley and Goldberg, 2003; Leathers and Olson, 2012). Modulation of LIP neurons by attention, reward, and expectation has been vastly studied (Gottlieb et al., 2014); however, in many neurophysiological experiments a clean dissociation between reward and attention was not provided (Maunsell, 2004). Reward biases also affect BR dynamics (Marx and Einhäuser, 2015). Although neural signals in LIP co-vary with the animal’s final decision, they are also modulated by the quality of the sensory evidence (Shadlen and Newsome, 1996, 2001) and informational properties of visual cue, like novelty, independent of the reward associations (Foley et al., 2014). Mere stimulus-reward associations can modify salience, or the ability of a stimulus to bias attention (Peck et al., 2009); an observation which is supported by psychophysical evidence in humans (Anderson et al., 2011). Manipulation of reward in a behavioral task could have implications in many cognitive processes such as decision making, task-reward association (Wisniewski et al., 2015), and attention (Peck et al., 2009).

Here, we investigated the possible role of the LIP of the rhesus macaque in perception and perceptual transitions using single cell electrophysiology and binocular flash suppression (BFS; Wolfe, 1984), a visual stimulation paradigm that can dissociate the sensory input from subjective perception. More specifically, during BFS, two sufficiently different visual images are presented asynchronously to the two eyes resulting to the complete perceptual suppression of the initially presented image and the predictable sole perception of the later appearing one (for more details see section “Materials and Methods”). Notably, the initially presented image is persistently presented to one of the eyes while the subject perceives the image presented to the contralateral eye. Thus, BFS ensures excellent control over the subject’s perceptual state, and unlike BR, the subjective report is not mandatory. Importantly, a number of studies demonstrated that the BFS phenomenon goes beyond simple sensory adaptation to the first stimulus (Keliris et al., 2010).

Moreover, in a second experiment we characterized LIP responses to BFS in the presence of reward asymmetry that was introduced between the two competing stimuli during training sessions before the recordings. We found that in comparison to the responses acquired with equal rewards, LIP demonstrated increased responses and enhanced magnitude of stimulus and perceptual selectivity.

## Materials and Methods

### Ethics Statement

The experimental and surgical procedures were performed with great care and were fully compliant with the guidelines of the local authorities (Regierungspräsidium Tübingen, protocol Nr. KY1/02), the European Community guidelines for the care and use of laboratory animals (EUVS 86/609/EEC), and the recommendations of the Weatherall report. The regional authorities (Regierungspräsidium Tübingen) approved our experimental protocol (Nr. KY1/02) and the institutional representatives for animal protection supervised all procedures. Animals were kept in large cages located adjacent to the training and experimental facilities. Space in these cages allows swinging and jumping, and enrichment equipment such as toys were changed frequently. Group housing was maintained to increase the quality of life by rich visual, olfactory, auditory and social interaction and stimulation for play. Balanced nutrition and regular veterinary care and monitoring were provided. Chamber implantation and an anatomical scan were performed while the animals were under general anesthesia and aseptic conditions. To alleviate post-surgical pain, we administered analgesics for a week after the surgery (also see surgical procedures below).

### Subjects and Surgical Procedures

Two adult male rhesus monkeys (*Macaca mulatta*) with code names C07 and D10, aged 8 and 6 years, weighing 9 and 11 kg, respectively were used in the experiments. Medical-grade titanium recording chambers were positioned according to stereotaxic coordinates over the intraparietal sulcus (IPS) of the right hemisphere in both monkeys. This was aided by high-resolution magnetic resonance anatomical imaging. Dimensions and parameters of the skull extracted from these scans were used for designing the head-posts and the recording chambers to fit the skull surface. The anatomical scan and recording chamber implantation were done in separate interventions while the animals where under general anesthesia and aseptic conditions. A more detailed description of these methods can be found elsewhere (Keliris et al., 2010). Recording from the LIP was confirmed by the histology performed on one of the animals (C07) after the experiments were finished. It both monkeys, recording from LIP was confirmed by careful examination of visual properties of recorded neurons according to typical LIP responses, at the beginning of each recording session and through audio and visual inspection. Also, the trajectory of penetration of tetrodes allowed us to confirm the location of the tip of the tetrode after passing several layers and tissues, including the dura, gray matter, white matter, and IPS.

### Data Acquisition

Extracellular recordings were done non-chronically with one or two manually adjustable, custom-made micro drives and twisted-wire tetrodes with the help of a grid system. Details have been described elsewhere (Tolias et al., 2007; Keliris et al., 2010). The recording chambers gave access to the LIP by penetrating area 5 perpendicularly and passing through medial intraparietal area (MIP) and the IPS. We advanced the tetrodes in the brain tissue after penetrating the dura by a guide-tube. Advancing the tetrode in the guide-tube by hand enabled us to have a vivid feeling of penetrating the sulcus. The physiological responses of different areas from the cortex beneath the dura until the target area in LIP was confirmed in every recording session. We stopped advancing the tetrodes as soon as we entered the LIP right after the sulcus and left them there fixed for approximately 1 h for the electrodes and the tissue to become stabilized. When a stable and reliable signal was acquired, we started the calibration procedures and data collection. The animals’ eye movements were monitored online with non-invasive infrared eye-tracker.

Multi-unit activity was sampled at 32 kHz, digitized, and stored using the Cheetah data acquisition system (Neuralynx) and was defined as the events that exceeded a predefined threshold (25 μV) of the filtered (600 Hz–6 kHz) and digitized signal. Following each threshold crossing, a segment of 32 samples (1 ms) was extracted from all four channels of the tetrode and these waveforms were stored for offline clustering. Single-unit spikes were then isolated from multiunit activity by a custom-built clustering system (Tolias et al., 2007) that uses features extracted from the stored multiunit spike waveforms.

### Visual Stimuli

A dedicated graphics workstation (TDZ 2000; Intergraph Systems) running an OpenGL-based program was used for rendering visual stimuli, while the behavioral aspects (e.g., juice reward, trial abortion) were controlled using the QNX real-time operating system (QNX Software Systems Ltd., Kanata, ON, Canada). The display system comprised of a custom-made mirror stereoscope with an LCD monitor (resolution of 1,024 × 768; refresh rate of 60 Hz) on each side and allowed for dichoptic presentation of stimuli.

Each session began with a calibration procedure to ensure that the monkeys could correctly overlay (fuse) the central fixation markers (0.2°) on the two displays. Thereafter, a coarse receptive field mapping was performed to position the stimuli for the experiments. The multi-unit responses were put through a sound amplifier (Grass Technologies) so that the experimenter could evaluate the gross location of the receptive fields and the preferences of the multi-unit responses towards different stimuli, locations and sizes as described previously (Keliris et al., 2010).

**Figure 1:**
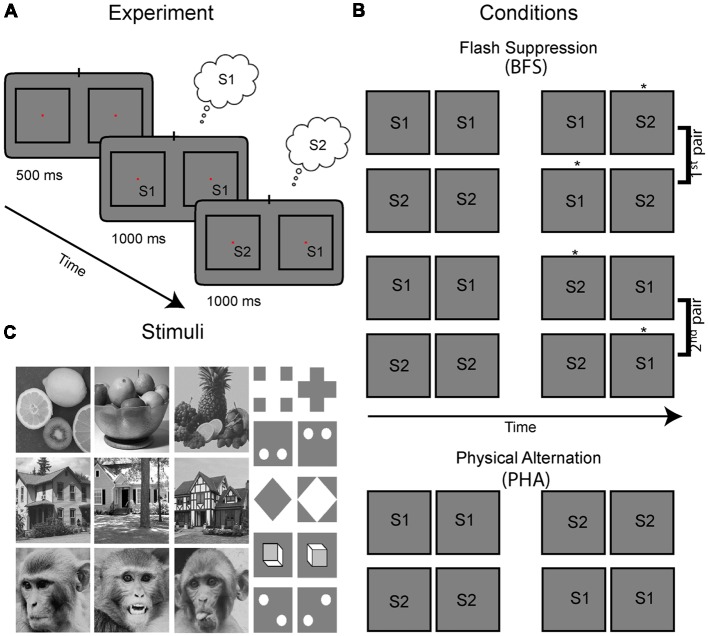
Experimental paradigm, visual stimulus conditions and stimuli. **(A)** Stimulus presentation for each trial and condition started with a fixation period with duration of 500 ms, followed with presentation of a congruent stimulus in homotopic retinal locations across the two eyes for 1 s. The left and right eyes viewed separate monitors *via* a stereoscope and the stimuli marked S1 and S2 were placed in the RF of the recorded neurons. In the cartoons presented here, the views of the two eyes are drawn in separate left-right squares. Then, the stimulus in either one [as shown in the example leading to incongruent stimuli in the two eyes; binocular flash suppression (BFS)—see percept noted in the small clouds] or both of the eyes (congruent; PHA) switched to a different image. During BFS this manipulation results in perceptual suppression of the previous image and thus perceptual transitions similar to the congruent stimulus switch in both eyes (PHA). **(B)** All possible combinations of stimulus pairs used in our experiments which included four BFS and two PHA conditions. Depending on the initial stimulus (S1 or S2) and in which eye(s) the switch was performed, a different percept is elicited during the last stimulus phase (the perceived stimulus is highlighted with an asterisk next in each panel). Note that the BFS conditions can be grouped in two pairs with exactly the same stimuli S1, S2 in the two eyes, albeit with different percepts. Control conditions are physical alternations (PHA), where both stimuli in two eyes switched to the new pattern. **(C)** The battery of stimuli we used consisted of natural as well as synthetic images.

First, a battery of natural and generic images from different categories including fruits, houses, monkey faces, and shapes were presented to the animal (Figure 1C). Then, two sufficiently different stimuli (S1, S2) were selected from separate categories (e.g., a house vs. a face) based on the elicited visual responses that were monitored online *via* auditory feedback as well as histograms that were updated online up to around 10 trials per stimulus. Our goal was to select stimuli with different response levels if present. If this was not possible, we selected randomly two images from different categories that elicited good visual responses.

### Experimental Design

To study the relationship between neural activity and perceptual modulations, we used the paradigm of BFS. In order to initiate the BFS trial, the monkeys had to first passively fixate for 500 ms on a central fixation point (0.2°) which appeared in the center of the screen. Immediately after, while the monkeys continued to fixate, one of the selected images (S1) was presented for 1 s to both eyes. Then, in BFS trials, the stimulus in one of the two eyes was switched to the second image (S2) resulting in interocular presentation of both stimuli (S1, S2) for another second (Figure 1A). It has been repeatedly shown previously (Brascamp et al., 2007), that during this stimulus phase the newly presented image (S2) is being invariably perceived. Thus, for the example trial presented in Figure 1A, the subject initially perceives S1 for 1 s and then his perception switches to S2 for another second even though image S1 is persistently presented (albeit perceptually suppressed) in the contralateral eye. Notably, the order of stimuli and eye of presentation of the second stimulus can be switched resulting in four different BFS conditions (Figure 1B). These conditions can be grouped in two pairs with identical stimuli during the incongruent stimulus phase (e.g., for the first two conditions S2 is in the left eye and S1 in the right—albeit with different percepts) and can thus dissociate stimulus from perception. Also important to note, the exact perceptual sequence of either S1 to S2 or S2 to S1 perceived during BFS trials can be simulated by physical alternation trials (PHA) in which the stimulus in both eyes is switched during the second stimulus phase (Figure 1B, bottom rows). Importantly, these trials do not have interocularly conflicting stimuli and can be used for identifying image selectivity during the two time periods.

#### Experiment 1

The stimuli were placed within the receptive fields of the recorded sites with approximate sizes of 4–6° based on short online mapping by moving bar/image stimuli (not recorded). For a trial to be considered successful the animal was required to maintain fixation within a window with a radius 1° from the center of the fixation marker throughout the duration of the trial. At the end of each successful trial, a drop of juice (~0.25ml) were delivered as a reward. If the animal broke fixation the trial was aborted, and no reward was delivered. Recording sessions included 10–200 (typically 50–100) trials per condition (i.e., for each of the four BFS conditions and the two PHA conditions in Figure 1B) and a pseudo-randomization procedure assured approximately equal trials across conditions over time.

#### Experiment 2

For experiment 2 all conditions were identical to experiment 1 except that one of two *a priori* selected stimuli was coupled with a triple amount of juice reward (3 drops/~0.75 ml) while the other stimulus was also rewarded albeit with less amount (1 drop /~0.25 ml). The stimulus-reward pairing took place in training sessions before the BFS recordings.

### Statistical and Data Analysis

We used custom programs written in MatLab (The Mathworks Inc., Natick, MA, United States) for data analysis. First, to identify the visual stimulus responsive neurons we performed a one-way anova that included the fixation window (−300 to 0 ms) and four different stimulus periods corresponding to the transient responses after stimulus onset (0–300 ms) or switch (1,000–1,300 ms) and the sustained responses of the first (500–800 ms) and second stimulus (1,500–1,800 ms). As we took the time average responses in these windows per trial, we chose windows of equal duration to avoid biases. Slight changes in the exact placement of these windows produced very similar results. A neuron was considered visually responsive and has been used for further analysis if we acquired data of at least 10 trials per condition and if it crossed the criterion of α = 0.05 with the probability P being Bonferroni corrected for multiple comparisons across all neurons tested and the six stimulus conditions that were tested with separate analyzes of variances (ANOVAs). These conservative criteria identified clearly the responsive neurons that were approximately 50% of all isolated recorded cells. To assess physical stimulus selectivity and perceptual modulations we first identified the neurons showing significant differences across the relevant conditions either during the transient (first 300 ms after stimulus onset or switch) or the sustained responses (post 300 ms after stimulus onset or switch) by using nonparametric *post hoc* wilcoxon rank sum (WRS) tests for equal medians that were Bonferroni corrected based on the number of tests. Then, the relevant pairs of conditions tested were sorted per neuron to preferred and non-preferred based on the mean activity during the relevant time-window (i.e., 1,000 ms either before or after the stimulus switch) and selectivity was quantified by using d-primes calculated with the following formula:

(1) $${\text{d}}^{\prime }=\frac{r_{\text{pref}}-r_{\text{npref}}}{\sqrt{(\sigma_{\text{pref}}^{2}+\sigma_{\text{npref}}^{2})/2}}$$

The d-primes were calculated initially per time point across neurons and then averaged over time and reported as <d′>_time_. To perform statistical comparisons of the selectivity across conditions, we performed paired *t*-tests (using the timepoints) across neurons.

## Results

We recorded extracellular activity from LIP in the right hemisphere of two rhesus macaques. Single cell activities were recorded during the paradigm of BFS for both experiment 1 (pre-reward manipulation) and experiment 2 (post-reward manipulation). In experiment 1 we recorded 28 sessions in monkey C07 (54 cells) and 37 in D10 (95 cells), while in experiment 2 we acquired data from 36 sessions in C07 (84 cells) and 34 in D10 (77 cells). In total, we recorded from 310 single cells and based on our criteria (see “Materials and Methods” section) 155 neurons (50%) were identified as visually responsive with sufficient number of trials per condition and were selected for further analysis. These consisted 74/149 (49.7%) of the cells recorded during experiment 1 and 81/161 (50.3%) of the cells recorded during experiment 2.

### Experiment 1

**Figure 2:**
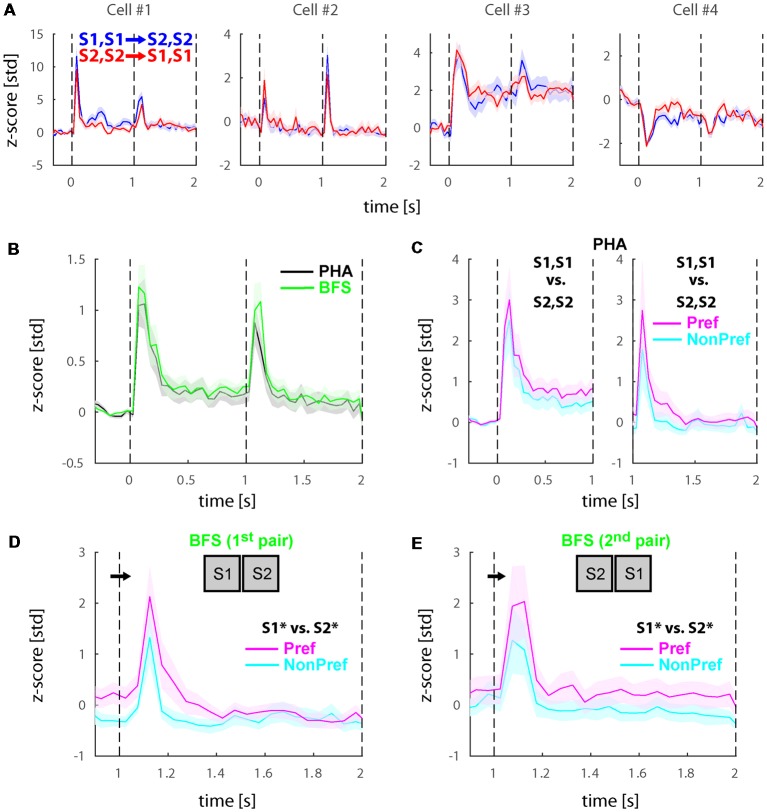
Physical and perceptual modulations the populations of selective neurons before reward manipulation (Experiment 1). **(A)** Responses during physical alternation conditions in four example lateral intraparietal area (LIP) neurons. Blue and red traces denote trials with switches from S1, S1 to S2, S2 and S2, S2 to S1, S1, respectively. Note that the relative magnitudes of the onset transients and the transients after the stimulus switch, the sustained responses, and sometimes the directions of the transient and sustained responses are different in these example cells. Also note that the selectivity across the two stimuli is generally weak. **(B)** Average responses across all visually responsive neurons (*N* = 74) collapsed for all PHA (black) and BFS (green) conditions. LIP neurons showed a transient response to stimulus onsets and after the stimulus switch for both types of trials. **(C)** Population response during PHA in only the neurons showing significant selectivity across the two stimuli (S1, S1 vs. S2, S2) for each phase of stimulus presentation (see section –“Materials and Methods”). In the left panel (*N* = 21), the significance and sorting of conditions were performed based on the mean responses in each selective neuron before the switch and plotted as preferred (magenta) and non-preferred (cyan). In the right panel (*N* = 10), the significance and sorting was performed according to the responses after the switch. **(D,E)** Population responses of perceptually selective cells in the two pairs of conditions presenting exactly the same stimuli across the two eyes (*N*_pair1_ = 9; *N*_pair2_ = 13). The sorting of preferred and non-preferred conditions in this case was based on the perceived stimulus S1* or S2*. All responses were normalized to z-scores based on the mean and standard deviation during the fixation baseline period. Solid lines and shaded areas represent the mean ± standard error of the mean (SEM) respectively of the responses across trials **(A)** and neurons **(B–E)**.

The recorded cells typically showed an initial burst of activity directly after stimulus onsets as well as after stimulus switches. Figure 2A presents the activity of four example cells during the physical alternation conditions [i.e., a binocular congruent stimulus (S1) was presented for 1 s and then switched to a second stimulus (S2) in both eyes; blue lines and shaded standard error of the mean (SEM)] compared with the reverse order of presentation (i.e., S2 presented first and switched to S1; red lines and shaded SEMs). The cells #1 and #2 were more typical examples showing a transient burst of activity directly after the presentation of stimuli, after switches or both with varying amplitudes. The other two cells showing both transient as well as sustained responses (#3) or deactivations (#4) were rarer. Note that all four cells demonstrated weak to no selectivity across the two stimuli both in the first second of presentation as well as after the switch (i.e., between 1 to 2 s from stimulus onset). To unravel if these effects were representative across the population, we plotted the average of all visually responsive cells (*N* = 74) in Figure 2B (black). We observed that the transient responses were present in the population with approximately equal amplitudes during stimulus onset and switch. Previous fMRI studies in humans reported low or no changes in the activity of fronto-parietal areas during physical stimulus changes such as the ones presented here, while stronger transient responses were found during perceptual alternations (Lumer et al., 1998; but also see Knapen et al., 2011). To investigate if the transients in cell activity would be larger during perceptual switches, we also plotted the population mean across the same cells during the BFS conditions (green). We found that PHA and BFS transients were not significantly different (WRS-test, *P* > 0.05).

Further, we investigated the selectivity of the cells to the two stimuli. For the physical alternation (PHA) condition, we found that 21/74 (28.4%) of cells demonstrated significant preference for one of the two stimuli during the first time-window of presentation (i.e., before the stimulus switch), while only 10/74 (13.5%) showed significant preferences after the switch (WRS-test, *P* < 0.05). To visualize these preferences, we sorted the two stimuli separately for each time window to preferred (Pref) and non-preferred (NonPref) and plotted the mean responses for the significantly selective cells before (*N* = 21) and after (*N* = 10) the stimulus switch in Figure 2C left and right panels, respectively. Similar to single cells, the population of selective neurons demonstrated weak selectivity as measured by d′ across the preferred and non-preferred stimuli for both time-windows of stimulus presentation (<d′_pre_>_time_ = 0.23, <d′_post_>_time_ = 0.21).

Last, we investigated whether cells would show differential activity during the incongruent BFS condition after the switch. Our stimulus design (see Figure 1) entailed two pairs of BFS conditions with exactly the same stimuli physically presented after the switch, albeit associated with different percepts (denoted as S1* or S2* but both stimuli are present) depending on the initial stimulus (i.e., always the newly presented stimulus is perceived after the switch). To this end, we performed statistical comparisons (WRS-test, *P* < 0.05) across these two pairs of conditions and found that 9/74 (12.2%) and 13/74 (17.6%) to be significant for the two pairs, respectively. In total (union operation) we found 20/74 (27.0%) of cells showing perceptual modulations. To visualize these preferences, we sorted the stimuli similarly to the physical alternation conditions (i.e., according to the activity of the perceived stimulus after the switch) and plotted the mean responses (Figures 2D,E). Similar to physical stimulus selectivity during PHA, the average perceptual selectivity of both BFS-condition pairs was weak (<d′_BFS_>_time_ = 0.28).

### Experiment 2

Inspired by some studies that demonstrated that the activity of LIP neurons is modulated by goal driven signals and value based decisions (Dorris and Glimcher, 2004; Sugrue et al., 2004, 2005; Kable and Glimcher, 2009), in this experiment we investigated if asymmetric reward across the two stimuli would boost the stimulus selectivity of LIP cells, and as a result potentially also increase their perceptual modulations. We paired one of the two stimuli with a bigger reward (3 drops of juice; ~0.75ml), while the other one was also rewarded albeit with a smaller reward (1 drop of juice; ~0.25ml). Importantly, the monkeys were presented with these stimulus-reward associations in training sessions that preceded the recordings of experiment 2 but after experiment 1 was finished.

**Figure 3:**
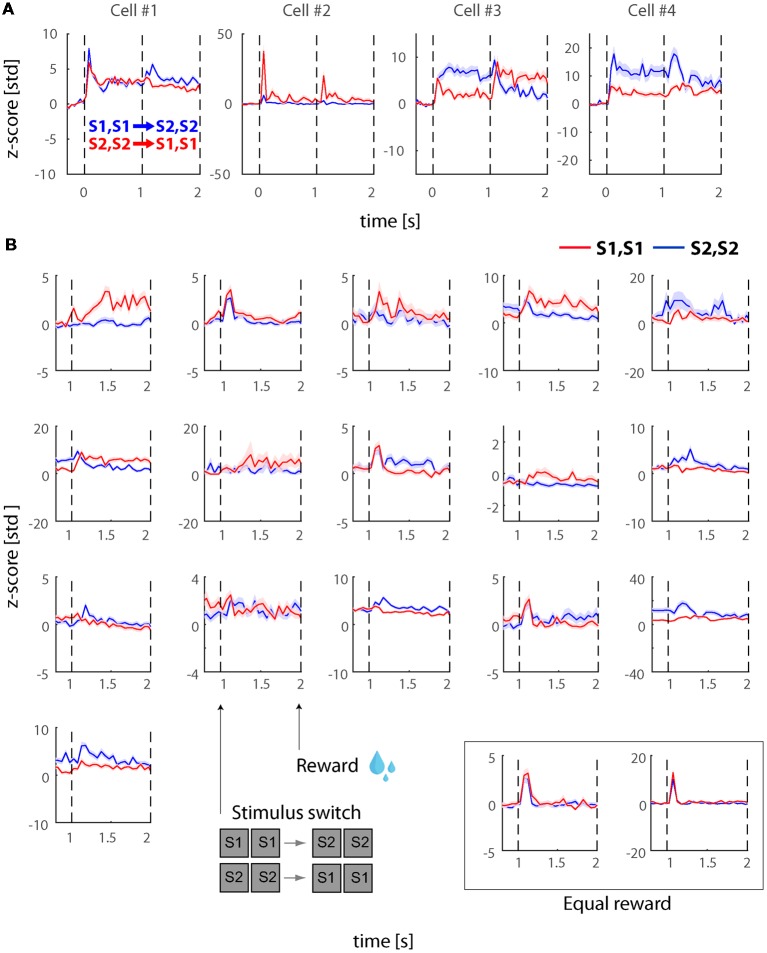
Physical stimulus selectivity in example cells post reward manipulation (Experiment 2). **(A)** Responses during physical alternation conditions in four example neurons after we introduced the asymmetric reward (larger reward for one of the stimuli). Blue and red traces denote trials with switches from S1, S1 to S2, S2 and S2, S2 to S1, S1, respectively. Note that the relative magnitudes of the onset transients and the transients after the stimulus switch, the sustained responses, and sometimes the directions of the transient and sustained responses are different in these example cells. Also note that the selectivity across the two stimuli is stronger in comparison to experiment 1 (see Figure 2A). **(B)** Neural responses during PHA after the switch. The figure demonstrates the cells (*N* = 16) that demonstrated significant stimulus selectivity during the last 300 ms of the 2nd time-window (i.e., just before asymmetric reward was delivered). In the inset at the bottom right we also plot the only two cells that showed significant differences during this period in experiment 1 (i.e., with equal rewards). Red and blue traces represent response to S1, S1 and S2, S2, respectively. All responses were normalized to z-scores based on the mean and standard deviation during the fixation baseline period. Solid lines and shaded areas represent the mean ± SEM, respectively of the responses across trials.

The recorded visually responsive cells during this experiment (*N* = 81), demonstrated similar dynamics to the cells recorded in experiment 1 with burst of activity during the onset and stimulus switches as well as some sustained response. However, we noted that some cells also demonstrated additional features we have not previously observed during experiment 1. More specifically, some of the cells demonstrated stronger and more sustained differences across the two stimuli. Figure 3A presents four example cells during the PHA conditions. As shown, some of these cells demonstrate sustained response differences during the first time-window of presentation but interestingly and even more consistently seem to show stimulus selectivity during the second time-window and until 2,000 ms after stimulus onset when the stimulus was removed and reward was provided to the subjects. To investigate this further, we tested how many cells show significant stimulus selectivity during the last 300 ms of stimulus presentation during this experiment and compared this to experiment 1. We found that 16/81 (19.8%) of cells showed selectivity during this phase in comparison to 2/74 (2.7%) of cells in experiment 1 (Figure 3B).

**Figure 4:**
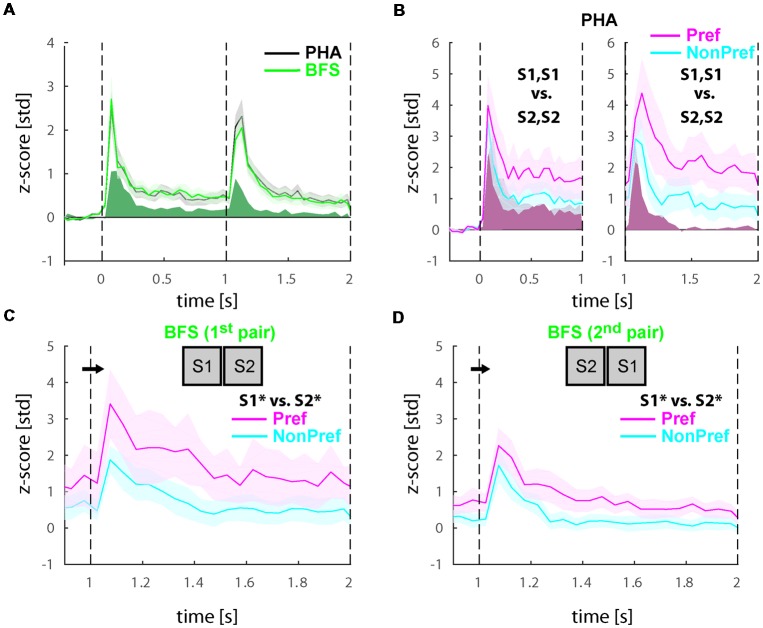
Physical and perceptual modulations in the populations of selective neurons after reward manipulation (Experiment 2). **(A)** Average responses across all visually responsive neurons (*N* = 81) collapsed for all PHA (black) and BFS (green) conditions. In solid green we also present the trace of the BFS conditions during Experiment 1 (see Figure 2B). As in Experiment 1, neurons showed a transient response to stimulus onsets and after the stimulus switch for both types of trials but response magnitude was higher. **(B)** Population response during PHA in only the neurons showing significant selectivity across the two stimuli (S1, S1 vs. S2, S2) for each phase of stimulus presentation (see section “Materials and Methods”). In the left panel (*N* = 22), the significance and sorting of conditions were performed based on the mean responses in each selective neuron before the switch and plotted as preferred (magenta) and non-preferred (cyan), while in the right panel (*N* = 16), the significance and sorting was performed according to the responses after the switch. **(C,D)** Population responses of perceptually selective cells in the two pairs of conditions presenting exactly the same stimuli across the two eyes (*N*_pair1_ = 16; *N*_pair2_ = 20). The sorting of preferred and non-preferred conditions in this case was based on the perceived stimulus S1* or S2*. All responses were normalized to z-scores based on the mean and standard deviation during the fixation baseline period. Solid lines and shaded areas represent the mean ± SEM respectively of the responses across neurons.

To investigate if the transient and sustained responses during experiment 2 were equivalent to those in experiment 1, we plotted the average of all visually responsive cells (*N* = 81) in Figure 4A (black and green for PHA and BFS, respectively). To allow comparison we also plotted (solid green) the average response of cells in experiment 1 which was presented in Figure 2B. We observed that transient as well as sustained responses were three-fold higher in experiment 2 (post-reward manipulation).

Further, we investigated the selectivity of the cells to the two stimuli. For the physical alternation, we found that 22/81 (27.2%) of cells demonstrated significant preference (WRS-test, *P* < 0.05) for one of the two stimuli before the switch and 14/81 (17.2%) after the switch. To visualize these preferences, we sorted the two stimuli for each significantly selective cell similarly to experiment 1 to preferred (Pref) and non-preferred (NonPref) for each window of stimulus presentation and plotted the mean responses before (*N* = 22) and after (*N* = 16) the switch in Figure 4B left and right panels, respectively. Interestingly, beyond the higher amplitude of neuronal activity (compare lines to solid red in Figure 4B), the population of significantly selective neurons for each time-window demonstrated slightly stronger stimulus selectivity between the preferred and non-preferred stimuli (<d′_pre_>_time_ = 0.30, <d′_post_>_time_ = 0.56) in comparison to experiment 1. Statistical analysis of the d′ values over time during the sustained response (pre: 350–950 ms; post: 1350–1950 ms; 12-time bins) using a paired one-tailed *t*-test across experiments (H_0_: d′_exp2_ > d′_exp1_) demonstrated a significant increase of selectivity both before *t*_(11)_ = 2.5756 *p* = 0.01 as well as after *t*_(11)_ = 4.3662 *p* = 0.00056 the stimulus switch. In particular the period after the stimulus switch which notably in temporal proximity to the reward demonstrated a higher increase relative to experiment 1.

Then, we investigated whether cells in experiment 2 would also show stronger perceptual selectivity in comparison to experiment 1 during the incongruent BFS conditions. First, we performed statistical comparisons (WRS-test, *P* < 0.05) across the two pairs of BFS conditions like we did in experiment 1 and found that 16/81 (19.8%) and 20/81 (24.7%) of cells showed significant differences between the percepts. In total (union operation) we found 29/81 (35.8%) of cells showing perceptual modulations. To visualize these preferences, we sorted the stimuli similarly to the physical alternation conditions (i.e., according to the activity of the perceived stimulus after the switch) and plotted the mean responses (Figures 4C,D). Similar to physical stimulus selectivity during PHA, the average perceptual selectivity of both BFS-condition pairs was stronger in experiment 2 in comparison to experiment 1 (<d′_BFS_>_time_ = 0.45). Statistical analysis of the d′ values over time during the sustained response (1350–1950 ms; 12-time bins) using a paired one-tailed *t*-test across experiments (H_0_: d′_exp2_ > d′_exp1_) demonstrated a significant increase of perceptual selectivity during experiment 2 (*t*_(11)_ = 5.5323, *p* = 0.000089).

**Figure 5:**
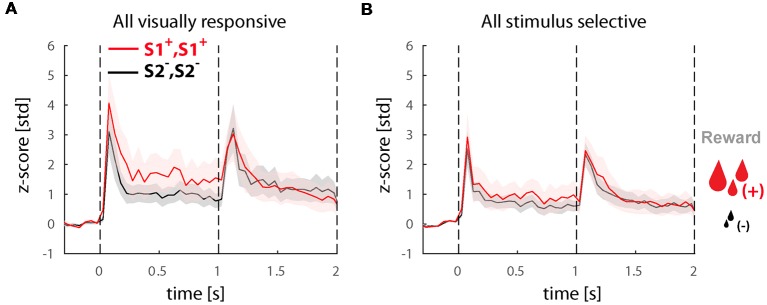
Responses in the population of selective cells in trials with large and small reward. **(A)** Population response of all visually responsive neurons (*N* = 81) during experiment 2. All conditions (i.e., BFS and PHA) were collapsed based on the stimulus before the switch S1^+^, S1^+^ leading to a large or S2^−^, S2^−^ leading to a small reward (note that perception always switches to the other stimulus after the switch in all conditions). **(B)** Population response of all visually selective cells (*N* = 22) also sorted for reward magnitude. All responses were normalized to z-scores based on the mean and standard deviation during the fixation baseline period. Solid lines and shaded areas represent the mean ± SEM, respectively of the responses across neurons.

Last, we wanted to know if the increased perceptual selectivity we found in experiment 2 is directly related to reward expectation. If this hypothesis is correct, the selectivity of neurons when plotted for the large vs. small reward trials is expected to be the same as the sorted preferred non-preferred stimuli. We performed this analysis both in the whole population of visually responsive cells (Figure 5A) as well as solely in the perceptually selective cells (Figure 5B). Surprisingly, we found that selectivity for reward size was (a) weaker than stimulus selectivity (<d′_pre_>_time_ = 0.25 in the in visually responsive cells and <d′_pre_>_time_ = 0.14 in the perceptually selective neurons) and (b) more pronounced during the first time-window of presentation before the switch (see <d′_pre_>_time_) in comparison to the second period when selectivity is virtually absent (<d′_post_>_time_ = −0.06 and 0.01) in the visually responsive and perceptually selective neurons, respectively. Note that the selectivity after the switch was significantly lower when compared with any of the stimulus or perceptual d′ in this experiment (paired *t*-tests, *p* ≪ 0.05).

## Discussion

In this study, we performed BFS experiments and recorded single cell electrophysiological signals in area LIP of the macaque. First, we showed that LIP neurons show transient responses to stimulus onsets and stimulus changes both during physical stimulus as well as perceptual switches with approximately the same amplitude. This result is consistent with a previous study in humans by fMRI that reported strong responses to stimulus switches during BR perceptual alternations (Lumer et al., 1998). However, the aforementioned study also claimed stronger transients during perceptual in comparison to physical stimulus exchanges while we found approximately equal responses (see Figures 2B, 4A). Given that our study was performed with a different technique (indirect BOLD fMRI measurements vs. single cell electrophysiology) and paradigm (BR vs. BFS) a number of reasons could explain this apparent discrepancy such as a difference between spontaneous and triggered alternations, the active reporting of subjective perception and the attention related to it. Moreover, a more recent fMRI study that better simulated the dynamics of BR during the physical alternations found results that are consistent with ours (Knapen et al., 2011). We further studied whether this transient response was affected by the reward manipulation. Interestingly, our asymmetric reward experiment could provide additional insights in respect to this discrepancy. We found that associating the stimuli with asymmetric rewards substantially enhanced (3-fold) the transients in the neuronal population across conditions (see Figure 4A). This could indicate that stronger attention coupled to the asymmetric value of the stimuli could potentially simulate the effects of the stochastic and thus unexpected perceptual transitions in BR.

The traditional approach to study the neural correlates of perceptual transitions in bistable phenomena is to capture the concurrence of activation in populations of neurons responding selectively to a particular stimulus and the subjective perception. In other words, when a neuron responds selectively to two stimuli during congruent presentation (i.e., higher responses to stimulus 1 vs. 2), it is also expected to show the same selectivity during incongruent presentation of both stimuli depending on which stimulus is perceived (i.e., without changes in the visual input the responses will increase and decrease during perception of stimulus 1 and 2, respectively). These neurons are then labeled *perceptually selective* to differentiate them from cells that only modulate their activity with changes in the sensory input. A number of studies from our laboratory and others have employed this approach in the past in feature and object selective areas in the ventral stream (Logothetis, 1998; Leopold and Logothetis, 1999; Keliris et al., 2010) and found that the proportion of perceptually selective neurons increases from around 20% in primary visual cortex to over 90% in inferotemporal cortex. However, stimulus selectivity is known to be weaker in the dorsal stream (Lehky and Sereno, 2007). To employ a similar approach, in this study we employed a battery of visual stimuli and tried to select those that showed differential responses for each experiment. We have found that with these stimuli 28.4% of cells in LIP demonstrated weak but significant stimulus selectivity during congruent stimulus presentation. During incongruent stimulus presentation across the two perceptions the percentage of modulated cells was lower (17.6%). This result together with our finding of strong transient responses indicated that LIP could be more associated to perceptual transitions rather than the perception of stimulus identity as have been also speculated in the past (Lumer et al., 1998).

To more absolutely settle this issue, we also performed a second experiment in which we included a differential reward association between the two presented stimuli (large and small reward at the end of the trial). Responses in LIP cells scale monotonically with the value of a planned saccade, which suggests the neural correlates of process of choices or representation of value before a choice in this area. LIP also encodes an abstract representation of the relative desirability of external stimuli apart from any specific motor plan (Dorris and Glimcher, 2004; Leathers and Olson, 2012). Thus, we speculated that the reward manipulations we employed could potentially increase the stimulus selectivity and provide a better tag to study perceptual modulations. We found that the percentage of neurons responding selectively to the stimuli remained similar (27.2%) to experiment 1 (i.e., before reward manipulation; 28.4%). However, the stimulus selectivity amplitude in these neurons was higher during experiment 2 (post-reward manipulation) in particular during the second phase of stimulus presentation after the transition (see Figures 3B, 4B). As congruent stimulus selectivity increased, we then tested if the neuros would respond more selectively to perceptual switches during BFS. We found that 24.7% of cells showed significant perceptual modulations, which is slightly higher than what we found before the reward manipulation (17.6%). The amplitude of perceptual modulations was also larger (see Figures 4C,D). Taken together, these results imply a role of stimulus-reward interaction in modulating perceptual selection but the contribution of area LIP in terms of the proportion of neurons remains low. This finding is consistent with previous studies that demonstrated similarly low proportions of selective neurons in LIP (Fanini and Assad, 2009; Romero et al., 2014; Subramanian and Colby, 2014) and corroborate the hypothesis that this area is more functionally relevant to attentional selection and decision making.

To better understand how reward modulates LIP neuron selectivity to the stimuli we have also plotted the responses of all conditions associated with a larger vs. a smaller reward. Surprisingly, we found that the larger reward was not always associated with the preferred stimulus neither in the whole population of visually selective cells (see Figure 5A) nor in the perceptually selective population (see Figure 5B). Given that the proportion of visually selective neurons before and after reward manipulation remained the same, we conjecture that reward does not actually change the visual stimulus selectivity, which is probably determined by the neuron’s connectivity and inputs, but rather is only able to modulate is amplitude. In terms of the perceptual modulations we identified, this corroborates that they do reflect direct correlates to stimulus perception rather than unspecific effects related to reward expectation.

We have previously shown that single-cell activity in V1 correlates with perceptual state (Keliris et al., 2010). The magnitude of perceptual modulations, however, is small in V1 compared to the higher visual areas (Logothetis and Schall, 1989; Leopold and Logothetis, 1996; Logothetis, 1998). Imaging studies in humans, on the other hand, found that perceptual suppression strongly modulates BOLD activity in primary visual cortex (Polonsky et al., 2000; Tong and Engel, 2001). In a more recent study, we suggested that these significantly bigger modulations may be due to the attentional demand and/or engagement of human subjects in a task (Bahmani et al., 2013, 2014). LIP is located a few synapses away from V1 (Ferrera and Grinband, 2006), and is massively interconnected with multiple visual areas including prefrontal cortex (Andersen et al., 1985, 1990; Cavada and Goldman-Rakic, 1989; Stanton et al., 1995; Lewis and Van Essen, 2000). The current study provides evidence that stimulus-reward associations in non-human primates passively fixating without active engagement in perceptual reports could increase stimulus and perceptual selectivity in LIP. Given that LIP has been strongly associated to attentional modulations *via* feedback to areas earlier in the cortical hierarchy, these signals could contribute to potential increases of perceptual modulations in earlier areas such as those observed in humans.

## Author Contributions

HB and GK conceived and designed the study, analyzed the data, and wrote the manuscript. HB and QL performed the experiments. NL supported the study, provided experimental equipment. All authors interpreted the experimental results and contributed to the final manuscript and gave final approval for publication.

## Conflict of Interest Statement

The authors declare that the research was conducted in the absence of any commercial or financial relationships that could be construed as a potential conflict of interest.
